# Prognostic and immunological value of *LINC01615*-related genes in kidney clear cell carcinoma: Integrated bioinformatics analysis and expression validation

**DOI:** 10.1097/MD.0000000000050176

**Published:** 2026-08-21

**Authors:** Shijie Guo, Shuangqin Xu, Peng Song, Jinshan Fu, Shengxing Wang, Wengui Xie, Yubin Wang

**Affiliations:** a Department of Urology, the First Affiliated Hospital of Hainan Medical University, Hainan, China; b Emergency Department, the First Affiliated Hospital of Hainan Medical University, Hainan, China.

**Keywords:** immune infiltration, immunotherapy, kidney renal clear cell carcinoma, *LINC01615*, risk model

## Abstract

*LINC01615*, a long noncoding RNA, plays a pivotal role in the progression of kidney renal clear cell carcinoma (KIRC). This study aimed to assess the prognostic value of *LINC01615*-associated genes in KIRC by developing a risk model. Differential expression analysis in the The Cancer Genome Atlas-KIRC dataset identified differentially expressed genes between high and low *LINC01615* expression groups, as well as between KIRC and control groups. Signature genes were subsequently selected through protein-protein interaction (PPI) network analysis, while prognostic genes were identified *via* Cox regression. The risk model was then constructed and validated using the E-MTAB-1980 dataset. Furthermore, an independent prognostic analysis identified key risk factors, and a nomogram was created for clinical application. Additional analyses, including enrichment analysis, immune-related analysis, drug sensitivity evaluation, and regulatory network construction, were performed to explore the underlying mechanisms in high and low-risk groups. The GSE40435 dataset was employed for the validation of prognostic gene expression. Reverse transcription-quantitative PCR (RT-qPCR) was conducted to confirm the expression levels of prognostic genes and *LINC01615* in clinical samples. *LINC01615* expression was found to differ significantly between KIRC and control groups, with notable survival differences observed between high and low expression groups. A total of 757 candidate genes were identified. Among these, *COL4A4*, *COL5A1*, and *COL15A1* were screened as prognostic genes, and a risk model with better accuracy was constructed. Age and risk score were recognized as independent risk factors, and the nomogram demonstrated enhanced predictive accuracy. Twelve drugs showed a significant negative correlation with risk scores. Additionally, the high-risk group exhibited an increased likelihood of immune escape. A regulatory relationship between hsa-miR-3163 and *COL4A4*/*LINC01615* was identified. In both The Cancer Genome Atlas-KIRC and GSE40435 datasets, *COL5A1* and *COL15A1* were overexpressed in the KIRC group. RT-qPCR results for *COL5A1* and *COL4A4* were consistent with the above findings, while *COL15A1* showed no significant differences in clinical samples, possibly due to the small sample size. *COL4A4*, *COL5A1*, and *COL15A1* were identified as prognostic biomarkers through bioinformatics analysis. The developed risk model offers valuable insights for clinical prognostic prediction and immunotherapy in KIRC.

## 1. Introduction

Renal cell carcinoma (RCC), one of the most prevalent urological malignancies, accounts for approximately 2%-3% of all cancer-related deaths and is showing an increasing incidence.^[1]^ Kidney clear cell carcinoma (KIRC) is the predominant histological subtype, constituting about 70%-85% of RCC cases.^[2]^ Early-stage KIRC often presents with few or no symptoms. However, as the tumor progresses, patients may experience hematuria, lumbar pain, and abdominal masses. Additionally, nonspecific symptoms such as weight loss, fever, and hypertension may also manifest in some patients.^[3,4]^ Despite advances in treatment strategies, including novel drugs and therapeutic regimens,^[5]^ KIRC still carries a high mortality rate due to its insidious onset, resistance to radiotherapy and chemotherapy, and propensity for metastasis. Particularly in advanced or metastatic cases, the 5-year overall survival (OS) rate is approximately 10%.^[6]^ Early detection, diagnosis, and intervention remain crucial for improving patient outcomes. Therefore, identifying highly specific biological markers is essential for early diagnosis and the guidance of personalized therapies for patients with KIRC.

Long noncoding RNAs (lncRNAs) are noncoding RNA molecules longer than 200 nucleotides.^[7]^ Although lncRNAs do not encode proteins, they play essential roles in gene regulation, including modulating gene activation, silencing,^[8]^ and alternative splicing.^[9]^ LncRNAs act as key regulators in cancer development and metastasis, while also serving as biomarkers and therapeutic targets for various diseases.^[10–12]^ For example, *LINC01615* has been shown to be involved in lung adenocarcinoma progression by regulating key Epithelial-mesenchymal transition (EMT) transcription factors.^[13]^ It also promotes breast cancer metastasis through the regulation of SIPA1,^[14]^ and influences hepatocellular carcinoma metastasis by modulating the extracellular matrix (ECM).^[15]^ Additionally, *LINC01615* plays a role in colon cancer development by regulating relevant gene expressions.^[16]^ Recently, a derroptosis-related lncRNA model, including *LINC01615*, has been proposed as a potential prognostic tool for KIRC.^[17]^ However, the precise mechanisms underlying the biological processes it regulates remain unclear. Consequently, investigating the prognostic significance of *LINC01615*-related genes in KIRC, developing corresponding risk models, and predicting potential targeted therapies based on these models is of considerable importance.

This study utilized public data from the Cancer Genome Atlas The Cancer Genome Atlas (TCGA), Gene Expression Omnibus, and ArrayExpress databases to identify prognostic genes associated with *LINC01615* in KIRC through bioinformatics approaches. A prognostic risk model was constructed to predict the survival of patients with KIRC, followed by comprehensive analyses, including gene set enrichment analysis (GSEA), immune infiltration, and immunotherapy. These findings offer new perspectives for clinical prognosis prediction and immunotherapy strategies in KIRC.

## 2. Materials and methods

### 2.1. Data source

The TCGA-KIRC dataset, which includes 613 KIRC and 72 control tissue samples, was obtained from TCGA database (https://portal.gdc.cancer.gov/). Among these, 522 KIRC samples had survival information. Additionally, 101 KIRC tissue samples from the E-MTAB-1980 dataset were sourced from the ArrayExpress database (https://www.ebi.ac.uk/arrayexpress). The GSE40435 (GPL10558) dataset, consisting of 101 KIRC and 101 control tissue samples, was acquired from the Gene Expression Omnibus database (https://www.ncbi.nlm.nih.gov/geo/).

### 2.2. Evaluation of prognostic and diagnostic value of *LINC01615*

Initially, *LINC01615* expression differences between KIRC and control samples in the TCGA-KIRC dataset were analyzed using the Wilcoxon rank-sum test (*P* < .05). Subsequently, based on the optimal cutpoint of *LINC01615* expression, KIRC samples with survival data from the TCGA-KIRC dataset were classified into high and low *LINC01615* expression groups using the surv_cutpoint function in the survminer package (v 0.4.9).^[18]^ Kaplan–Meier (KM) survival curves for both groups were generated with the survival package (v 3.5-5).^[19]^ Survival differences in OS, disease-specific survival (DSS), and progression free interval (PFI) were assessed between the 2 groups using the log-rank test.

To evaluate *LINC01615*’s potential in distinguishing KIRC tumor tissues from normal tissues, a receiver operating characteristic (ROC) curve was plotted for *LINC01615* within the TCGA-KIRC dataset, and the area under the curve (AUC) was calculated using the survivalROC package (v 1.0.3.1).^[20]^ A higher AUC value indicates better diagnostic performance.

### 2.3. Differential expression analysis

Differentially expressed genes (DEGs) between the high and low *LINC01615* expression groups in KIRC samples with survival data were screened using the DESeq2 package (v 1.40.2) (|log_2_ fold change| > 1, *P*.adjust < 0.05).^[21]^ The results were visualized using the ggplot2 package (v 3.5.0)^[22]^ and the circlize package (v 0.4.15).^[23]^ Additionally, DEGs2 between KIRC and control samples in the TCGA-KIRC dataset were also identified and visualized similarly.

### 2.4. Functional enrichment analysis

Candidate genes were derived by taking the intersection of DEGs1 and DEGs2 using the eulerr package (v 7.0.0).^[24]^ Gene Ontology (GO) and Kyoto Encyclopedia of Genes and Genomes (KEGG) pathway analyses were performed on these candidate genes using the clusterProfiler package (v 4.2.2)^[25]^ (*P*.adjust < .05). The results were visualized using the ggpubr package (v 0.6.0)^[26]^ for GO analysis and the treemap package (v 2.4-4)^[27]^ for KEGG pathway analysis.

### 2.5. Protein-protein interaction network construction

To investigate interactions among candidate genes, a protein-protein interaction (PPI) network was constructed using the Search Tool for the Retrieval of Interacting Genes/Proteins database (https://cn.string-db.org/). Genes with confidence scores >0.4 and confirmed interaction relationships were retained. The significant gene clusters were identified using the molecular complex detection plugin in Cytoscape software (v 3.1.1).^[28]^ The resulting PPI network of the signature genes was visualized in Cytoscape.

### 2.6. *LINC01615*-related prognostic genes screening

For prognostic gene identification, signature gene data from KIRC samples with survival information in the TCGA-KIRC dataset were subjected to univariate Cox regression analysis (hazard ratio (HR) ≠ 1, *P* < .05), proportional hazards (PH) assumption testing (*P* > .05), Least Absolute Shrinkage and Selection Operator (Lasso) regression analysis, and multivariate Cox regression analysis (HR ≠ 1, *P* < .05). The results of Cox regression analyses were visualized using forest plots. Lasso regression was performed using the glmnet package (v 4.1-6).^[29]^

### 2.7. Construction and validation of risk model

Based on the prognostic genes identified within the TCGA-KIRC dataset, a risk model was constructed using the following formula:


$$risk\;score=\sum_{i=1}^{n}coef\left(gene_{i}\right)\times expr\left(gene_{i}\right)$$

Using the obtained prognostic risk scores, samples were divided into high- and low-risk groups, with the median risk score as the cutoff. A risk curve was plotted, and a heatmap was generated to visualize the expression of prognostic genes across the 2 risk groups within the TCGA-KIRC dataset. KM survival curves for the 2 risk groups were generated using the survminer package (v 0.4.9), and survival differences were assessed using the log-rank test (*P* < .05). To evaluate the model’s performance, ROC curves were plotted for 1, 3, and 5 years, and the AUC was calculated. To further assess the accuracy and generalizability of the risk model, it was validated in the E-MTAB-1980 dataset.

### 2.8. Independent prognostic analysis

To identify independent risk factors, variables including age, grade, gender, tumor stage, T_stage, N_stage, M_stage, and risk score underwent univariate Cox analysis (HR ≠ 1, *P* < .05), PH assumption testing (*P* > .05), and multivariate Cox analysis (HR ≠ 1, *P* < .05).

The rms package (v 6.7-0) was used to score the independent risk factors, and the total score was computed by summing the scores of each factor.^[30]^ Mortality predictions for 1, 3, and 5 years were based on the total score, with higher scores corresponding to higher mortality rates. The results were visualized in a nomogram, and the corresponding calibration curve, ROC curves and decision curve analysis (DCA) curves were plotted.

### 2.9. Gene set enrichment analysis

To identify relevant pathways and biological mechanisms between the 2 risk groups, differential analysis was conducted using the DESeq2 package (v 1.40.2) on the TCGA-KIRC dataset. The log_2_ fold change values from the differential analysis were sorted from largest to smallest, which were then used as a sorting criterion for further analysis. GSEA was performed using the clusterProfiler package (v 4.2.2) (*P*.adjust < .05), with the KEGG pathway background gene set, “c2.cp.kegg.v2023.1.Hs.symbols,” obtained *via* the msigdbr package (v 7.5.1).^[31]^

### 2.10. Immune infiltration analysis

To explore variations in 28 immune cell types^[32]^ between the 2 risk groups, single-sample GSEA (ssGSEA) from the GSVA package (v 1.48.3)^[33]^ was employed to compute the immune cell scores for each sample in the TCGA-KIRC dataset. Stacked and box plots of the 28 immune cell scores across the 2 risk groups were generated using the ggplot2 package (v 3.5.0). The Wilcoxon rank-sum test was applied to identify immune cells with significant differences between the risk groups (*P* < .05). Spearman correlation analysis was performed on these immune cells and *LINC01615*-related prognostic genes using the psych package (v 2.2.9),^[34]^ with the results visualized by a correlation heatmap generated using the ggcor package (v 0.9.8.1).^[35]^ Additionally, Spearman correlation analysis was performed on the risk scores and these immune cells, and the resulting correlation heatmap was visualized using ggplot2.

### 2.11. Immunotherapy analysis

The 38 common immune checkpoints (ICs) were sourced from the literature.^[36]^ Expression differences of IC-related genes between the 2 risk groups in the TCGA-KIRC dataset were assessed *via* the Wilcoxon rank-sum test (*P* < .05). Box plots were plotted using ggplot2 to explore associations between the 2 risk groups and IC-related genes. Furthermore, tumor immune dysfunction and exclusion (TIDE) scores for the 2 risk groups were calculated *via* the TIDE official website (http://tide.dfci.harvard.edu/), and intergroup differences were compared using the Wilcoxon rank-sum test (*P* < .05) to predict potential immunotherapy responses.^[37]^

### 2.12. Drug sensitivity analysis

Based on the 198 chemotherapy/targeted therapeutic drugs available in the Genomics of Drug Sensitivity in Cancer database (https://www.cancerrxgene.org/), the 50% inhibitory concentration (IC_50_) values for these drugs in the case samples were calculated using the oncoPredict package (v 1.2).^[38]^ The IC_50_ differences between the 2 risk groups were assessed *via* the Wilcoxon rank-sum test (*P* < .05). Box plots were plotted to visualize the differences in IC_50_ values between the risk groups for the top 6 drugs with the most significant differences. Spearman correlation analysis was conducted to evaluate the relationship between risk scores and IC_50_ values for these drugs, and scatter plots were generated to visually represent the associations between risk scores and the top 3 drugs with the most significant IC_50_ correlations.

### 2.13. *LINC01615*-related prognostic genes regulatory network construction

To systematically investigate the complex regulatory interactions, prognostic gene-associated microRNAs (miRNAs) were predicted using the miRDB database (http://www.mirdb.org/) (filter condition: Target Score > 50) and the miRWalk database (http://mirwalk.umm.uni-heidelberg.de/) (filter condition: bindingp = 1). Shared miRNA-associated lncRNAs were subsequently identified using the StarBase database (http://starbase.sysu.edu.cn/). A regulatory network involving *LINC01615*, miRNAs, and mRNAs (prognostic genes) was then constructed.

### 2.14. Expression analysis and validation of *LINC01615*-related prognostic genes

The expression of *LINC01615*-related prognostic genes in TCGA-KIRC and GSE40435 datasets was examined, and differences in expression between KIRC and control samples were analyzed using the Wilcoxon rank-sum test (*P* < .05).

Additionally, reverse transcription-quantitative PCR (RT-qPCR) was performed to validate the expression of *LINC01615* and prognostic genes in clinical samples. This study was conducted in accordance with the Declaration of Helsinki, The Ethics Committee of the First Affiliated Hospital of Hainan Medical University approved the study. The patients/participants gave their written informed consent. Total RNA was isolated from 5 KIRC and 5 control tissue samples using TRIzol reagent (Ambion, USA). RNA purity was assessed by electrophoresis and NanoPhotometer N50. cDNA synthesis was performed using the SweScript First Strand cDNA Synthesis Kit (Servicebio, China), followed by Reverse transcription-quantitative RT-qPCR with Universal Blue SYBR Green qPCR Master Mix (Servicebio, China) on a CFX Connect RT-qPCR Detection System (BIO-RAD, USA). The primers for prognostic genes, *LINC01615*, and the internal reference gene (GAPDH) are provided in Supplementary Table 1, Supplemental Digital Content 1. The relative expression levels were calculated using the 2^-ΔΔCт^ method. Differences between KIRC and control groups were analyzed by t-test, with results presented using GraphPad Prism 5 (*P* < .05).

### 2.15. Statistical analysis

Bioinformatics analyses were performed in R, with Wilcoxon rank-sum tests and t-tests applied to assess intergroup differences, using a significance threshold of *P* < .05.

## 3. Results

### 3.1. *LINC01615* had better prognostic and diagnostic value in KIRC

**Figure 1.**
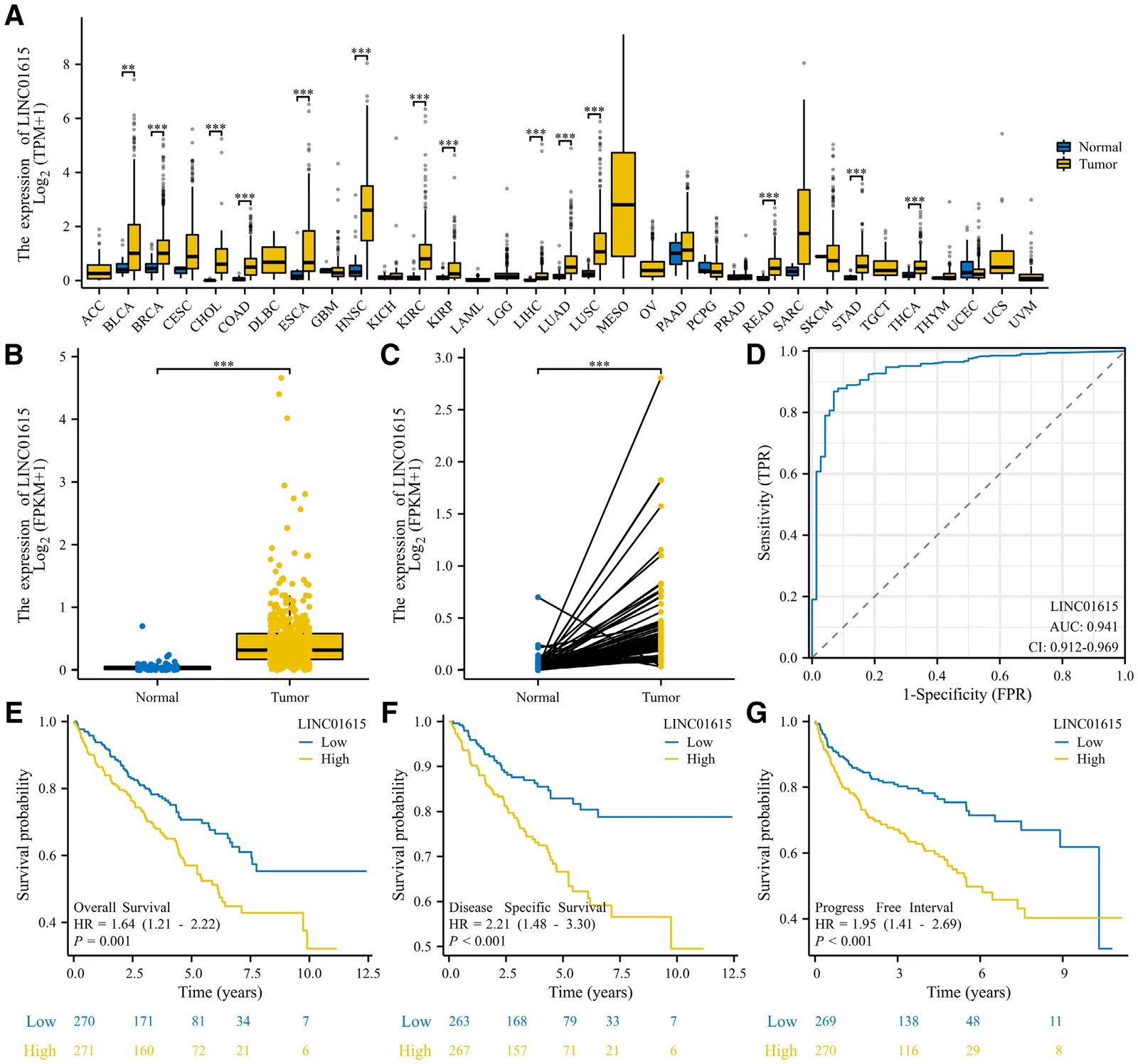
Expression of *LINC01615* in KIRC. (A) Expression of *LINC01615* in various cancers in the TCGA database; (B) Expression profile of *LINC01615* in normal kidney tissues and KIRC tissues; (C) Paired plot of *LINC01615* in 72 cases of normal kidney tissues and KIRC tissues; (D) ROC curve demonstrating the good diagnostic performance of *LINC01615*; (E–G) KM curves showing the association between *LINC01615* expression and OS, DSS, and PFI. *P < .05, **P < .01, ***P < .001

*LINC01615* exhibited high expression across various cancer types (Fig. 1A), with a significantly higher expression in KIRC samples compared to controls (*P* < .001) (Figs. 1B-C). The ROC curve analysis showed an AUC of 0.941 for *LINC01615* in KIRC, indicating its superior diagnostic performance (Fig. 1D). Survival analysis revealed a gradual decline in the survival of patients with KIRC over time, with the KM survival curve suggesting a higher survival rate in the group with lower *LINC01615* expression. Furthermore, OS, DSS, and PFI were significantly improved in this group (*P* < .05) (Figs. 1E-G), suggesting a correlation between elevated *LINC01615* expression and poor prognosis in KIRC.

### 3.2. Related functional pathways and PPI network of *LINC01615*-related candidate genes

**Figure 2.**
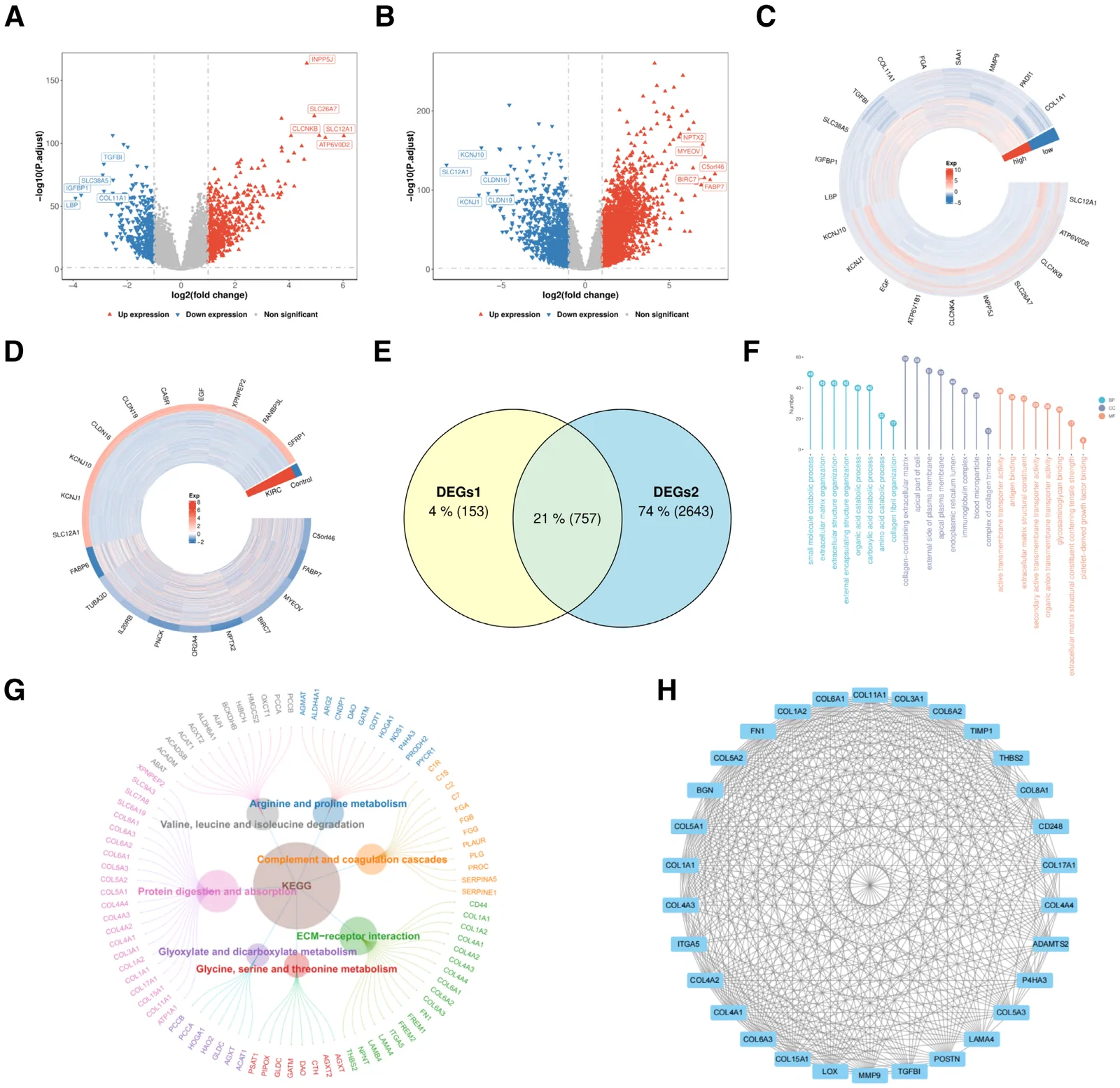
Screening of *LINC01615*-related DEGs. (A) Volcano plot and (C) heatmap of DEGs between the high and low *LINC01615* expression groups in KIRC samples. (B) Volcano plot and (D) heatmap of DEGs between KIRC samples and adjacent normal samples. Red indicates upregulated genes, while blue indicates downregulated genes in both the volcano plots and heatmaps. (E) Venn diagram showing the overlap between KIRC-related DEGs1 and DEGs2 between KIRC and normal controls. (F) Bar charts of GO enrichment analysis based on BP, CC, and MF. (G) KEGG pathway enrichment analysis. (H) PPI interaction network of *LINC01615*-related candidate genes. DEG = differentially expressed gene.

*LINC01615*-related candidate genes were first screened, identifying a total of 910 DEGs1, with 504 upregulated and 406 downregulated (Fig. 2A). A further 3400 DEGs2 were identified, including 2224 upregulated and 1176 downregulated genes (Fig. 2B). The expression heatmap depicted the top 10 most significantly up- and down-regulated genes (Figs. 2C-D). The intersection of DEGs1 and DEGs2 yielded 757 *LINC01615*-related candidate genes (Fig. 2E).

GO enrichment analysis was performed on these candidate genes, revealing significant enrichment in 289 distinct categories (Fig. 2F). This included 195 biological processes (BP) such as ECM organization, 36 cellular components (CC) like the collagen-containing ECM, and 38 molecular functions (MF) including active transmembrane transporter activity, antigen binding, and ECM structural constituent. KEGG pathway enrichment identified 24 significantly enriched pathways, including ECM-receptor interaction (Fig. 2G). These results suggest that *LINC01615*-related candidate genes are predominantly involved in these biological processes and pathways, providing insight into their potential roles in KIRC.

Following the removal of 156 independent targets, a PPI network was constructed for 30 key *LINC01615*-related candidate genes (Fig. 2H). This network consisted of 30 nodes and 357 protein interactions, highlighting the intricate connections between these genes and their potential significance in cellular functions and disease progression.

### 3.3. Prognostic genes were screened, and a risk model was constructed

**Figure 3.**
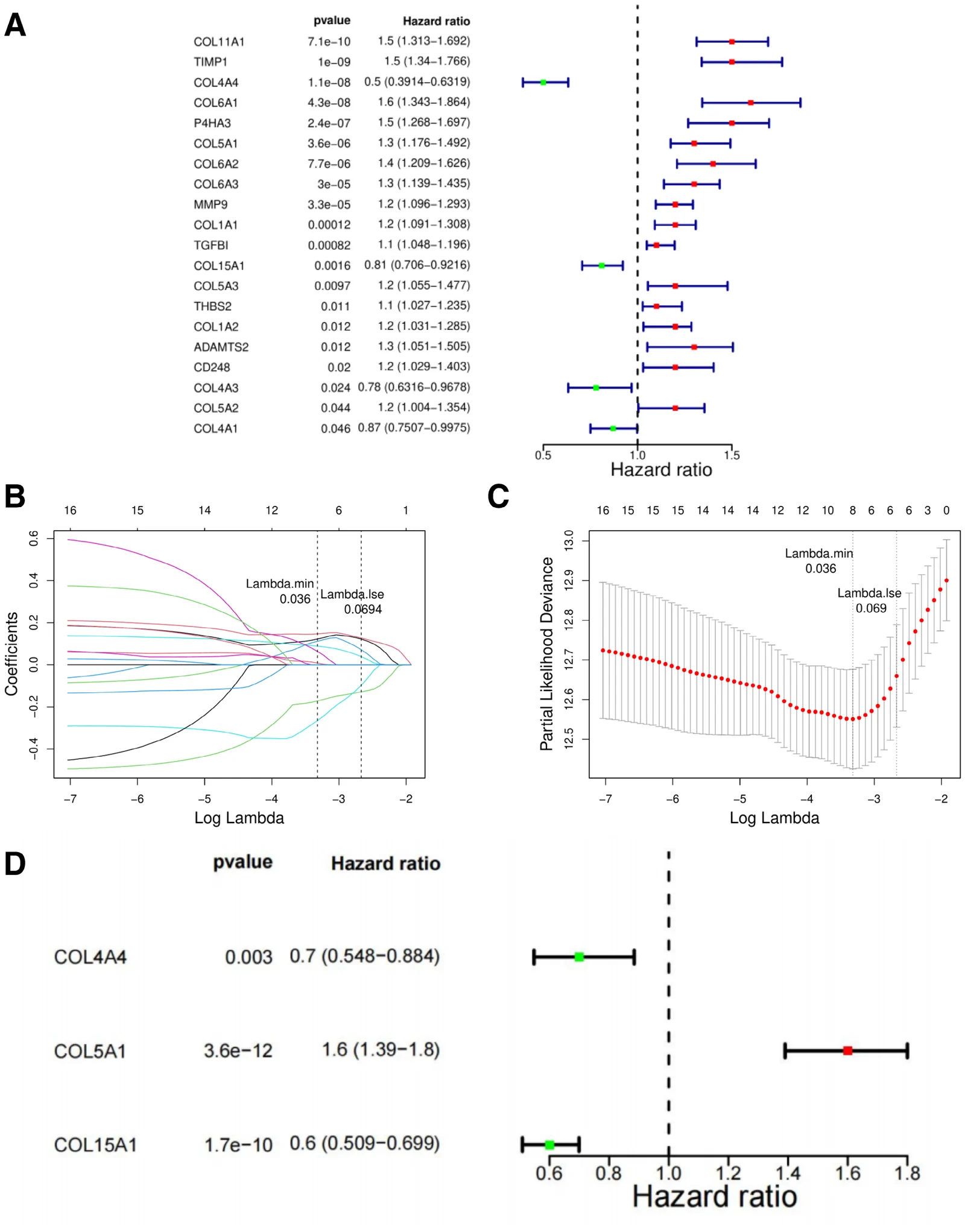
Screening of prognostic genes. (A) Results of univariate Cox analysis. (B) Results of LASSO regression analysis. (C) Cross-validation for adjusting the parameter selection in the LASSO Cox regression analysis. (D) Results of multivariate Cox analysis.

Univariate Cox analysis identified 20 genes (Fig. 3A), and the PH assumption test confirmed that *P* > .05 for these genes, satisfying the assumption. Lasso regression analysis reduced this list to 8 genes: *COL11A1*, *TIMP1*, *COL4A4*, *COL6A1*, *P4HA3*, *COL5A1*, *COL6A3*, and *COL15A1* (Figs. 3B-C). Multivariate Cox analysis of these 8 genes revealed 3 prognostic genes: *COL4A4*, *COL5A1*, and *COL15A1* (Fig. 3D).

### 3.4. Risk model was constructed

**Figure 4.**
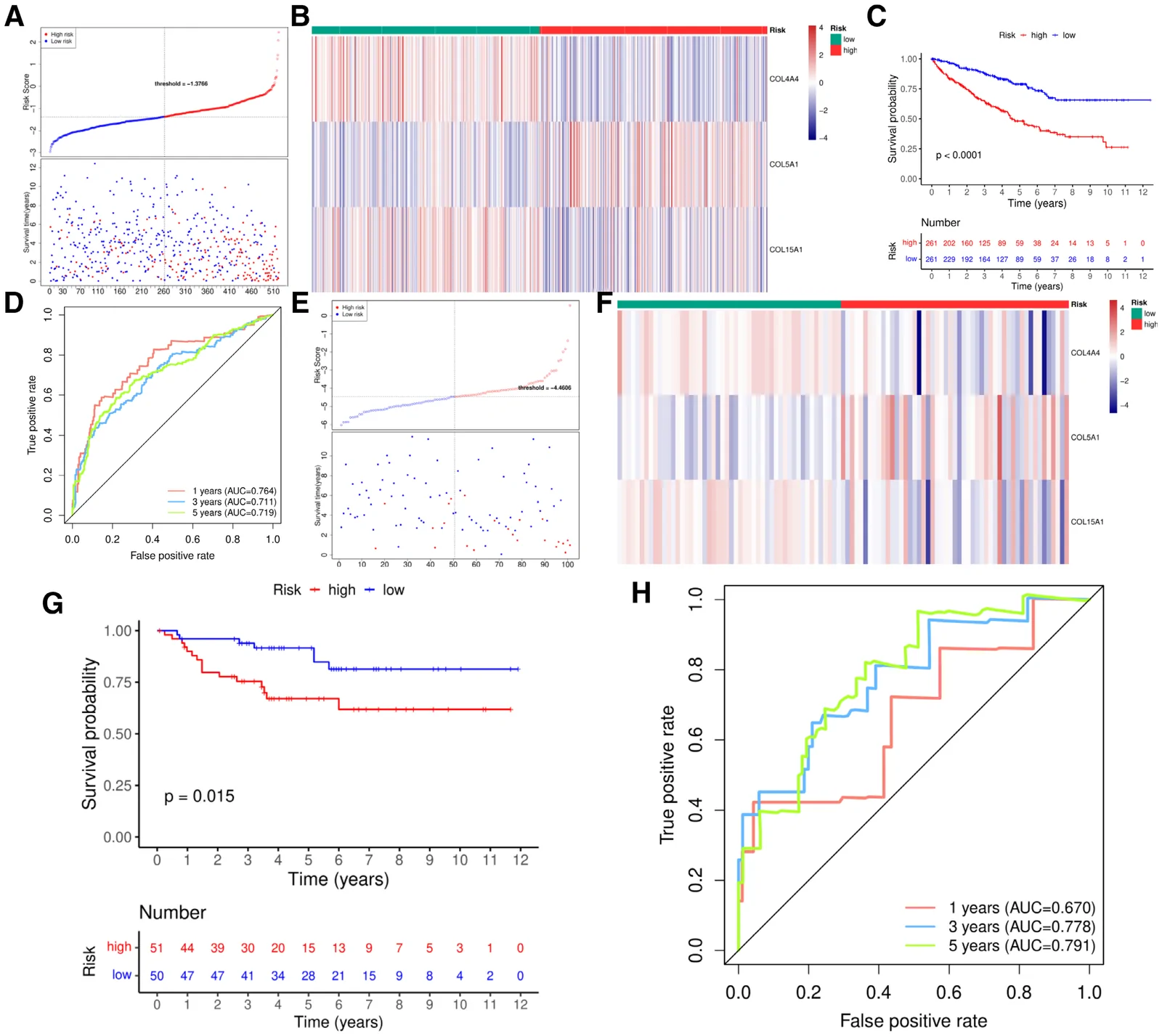
Establishment and validation of the prognostic gene risk model. (A) Distribution of patients based on risk scores from the TCGA-KIRC dataset. (B) Heatmap of the risk model based on risk scores from TCGA-KIRC dataset. (C) KM curves for the OS of patients in the high-risk and low-risk groups of the testing cohort. (D) ROC curve showing the predictive performance of the risk model in TCGA-KIRC dataset. (E–H) Distribution of patients based on risk scores, risk model heatmap, KM curves, and ROC curve in the GSE40435 dataset.

The risk model was developed for KIRC samples in the TCGA-KIRC dataset with survival data. The prognostic risk score model was defined as: risk score = (−0.3626) × *COL4A4* expression level + (0.4568) × *COL5A1* expression level + (−0.51,601) × *COL15A1* expression level. The risk curve demonstrated that the mortality rate was lower among patients with KIRC in the low-risk group (Fig. 4A). Furthermore, the expression of prognostic genes varied significantly between the 2 risk groups in the TCGA-KIRC dataset (Fig. 4B). Specifically, *COL4A4* and *COL15A1* expression was markedly higher in the low-risk group, while *COL5A1* expression was elevated in the high-risk group. This suggests that *COL4A4* and *COL15A1* may have a suppressive role in tumors at early or low-risk stages, while *COL5A1* might be associated with tumor progression or deterioration. The KM survival curve (Fig. 4C) revealed that patients with KIRC in the low-risk group had a significantly higher survival rate (*P* < .001). The AUC values for 1 year, 3 years, and 5 years were 0.764, 0.711, and 0.719, respectively (Fig. 4D), indicating the superior efficacy of the risk model.

To validate the model, it was tested using the E-MTAB-1980 dataset. The risk curve (Fig. 4E), gene expression patterns (Fig. 4F), KM survival curve (Fig. 4G), and ROC curve (AUC > 0.6) (Fig. 4H) confirmed the robustness and generalizability of the prognostic risk model. These results suggest that the model effectively stratifies patient risk levels and predicts survival outcomes, making it a valuable tool for personalized prognostic assessments in clinical practice.

### 3.5. Prognostic nomogram for KIRC based on independent risk factors

**Figure 5.**
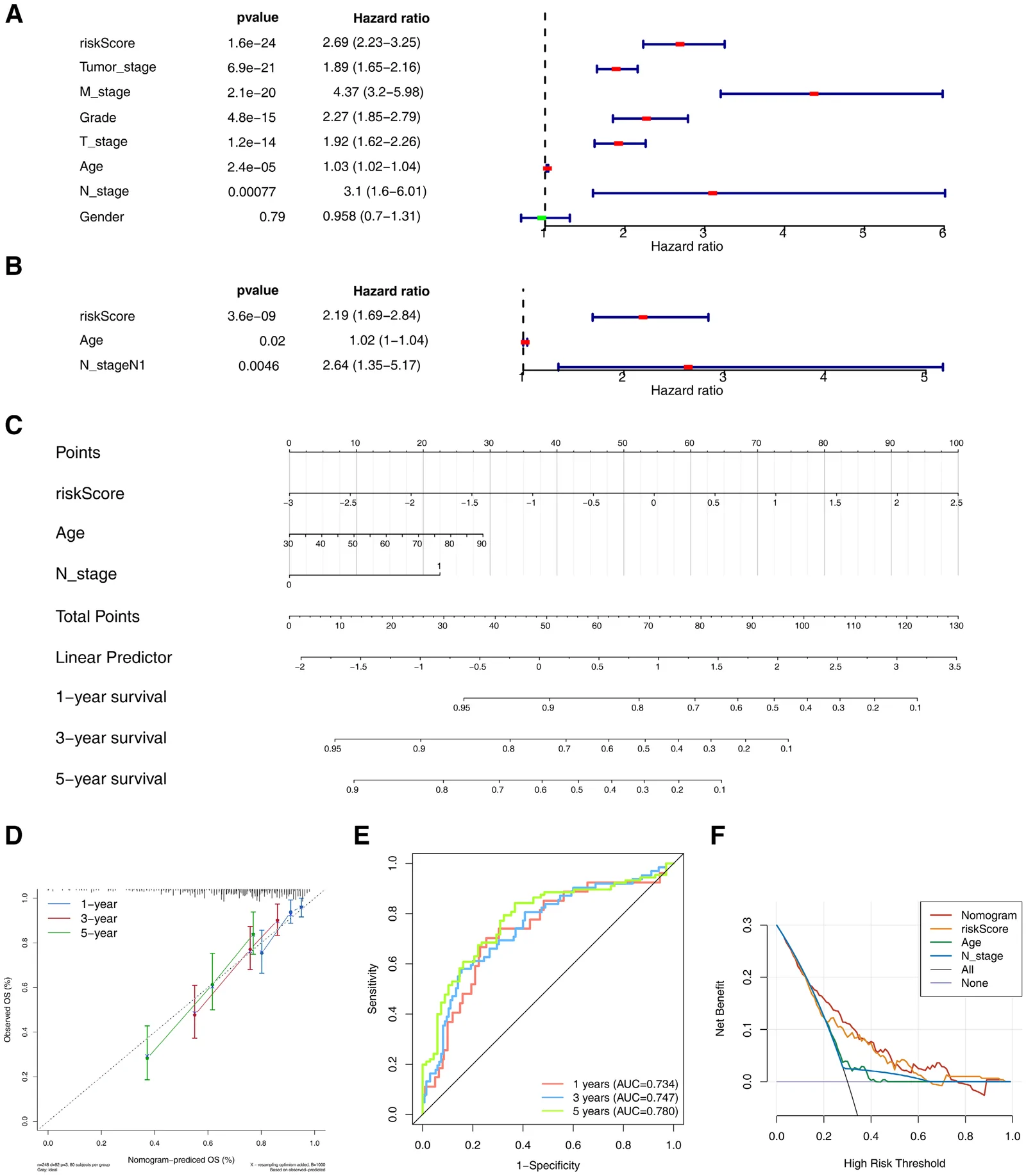
Independent prognostic analysis and nomogram establishment. (A) Results of univariate Cox regression analysis in the TCGA-KIRC dataset. (B) Results of multivariate Cox regression analysis in the TCGA-KIRC dataset. (C) Nomogram for predicting the 1-year, 3-year, and 5-year OS of patients with KIRC. (D) Calibration plots of the nomogram for 1-year, 3-year, and 5-year survival in the TCGA-KIRC dataset of KIRC patients and (E) its predictive performance. (F) The DCA suggests that the nomogram has higher practicality in clinical application.

Univariate Cox analysis identified key clinical characteristics: age, grade, tumor stage, T_stage, N_stage, M_stage, and risk score (Fig. 5A). The PH assumption test confirmed that age, N_stage and risk score met the hypothesis (*P* > .05) (Supplementary Figure 1, Supplemental Digital Content 2). Multivariate Cox analysis further retained age, N_stage and risk score as independent risk factors (Fig. 5B). The corresponding nomogram is shown in Figure 5C. The calibration curve exhibited a slope near 1, and the AUC was >0.7 (Figs. 5D-E), indicating that this model has strong predictive accuracy and can serve as an effective clinical tool. In addition, DCA further confirms that, compared with the use of any single independent prognostic factor alone, the nomogram demonstrates higher practicality in clinical applications (Fig. 5F).

### 3.6. Key pathways screening by GSEA

**Figure 6.**
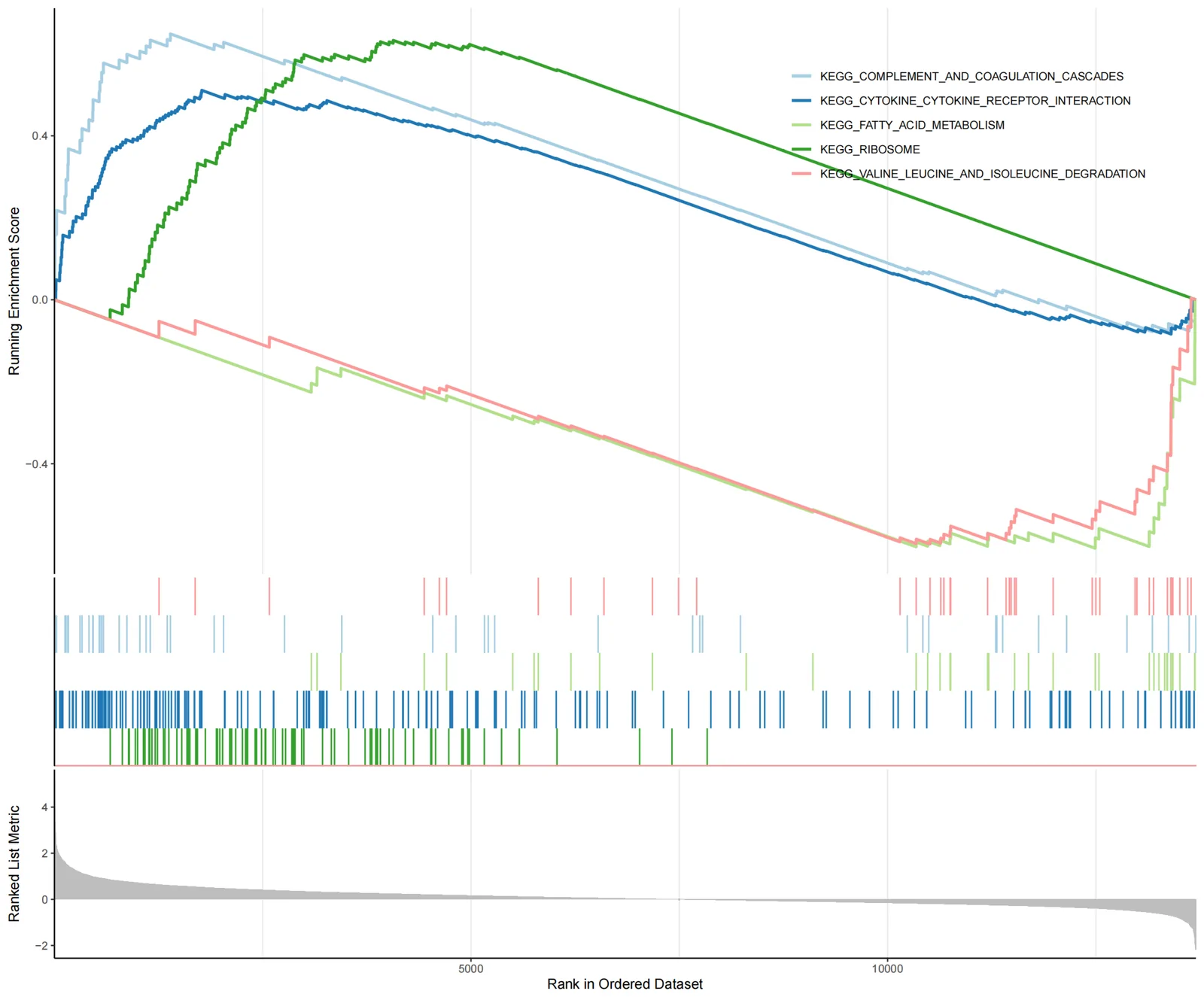
Key pathways screened by GSEA. KEGG = Kyoto Encyclopedia of Genes and Genomes.

Finally, GSEA revealed 31 significantly enriched pathways, including fatty acid metabolism (Fig. 6), which may be crucial in KIRC development and progression. These metabolic pathway enrichments reflect the universal energetic metabolic reprogramming characteristic of clear cell RCC. Targeting these pathways could provide novel therapeutic strategies and potential targets for KIRC treatment.

### 3.7. Difference in immune cell infiltration and immune response

**Figure 7.**
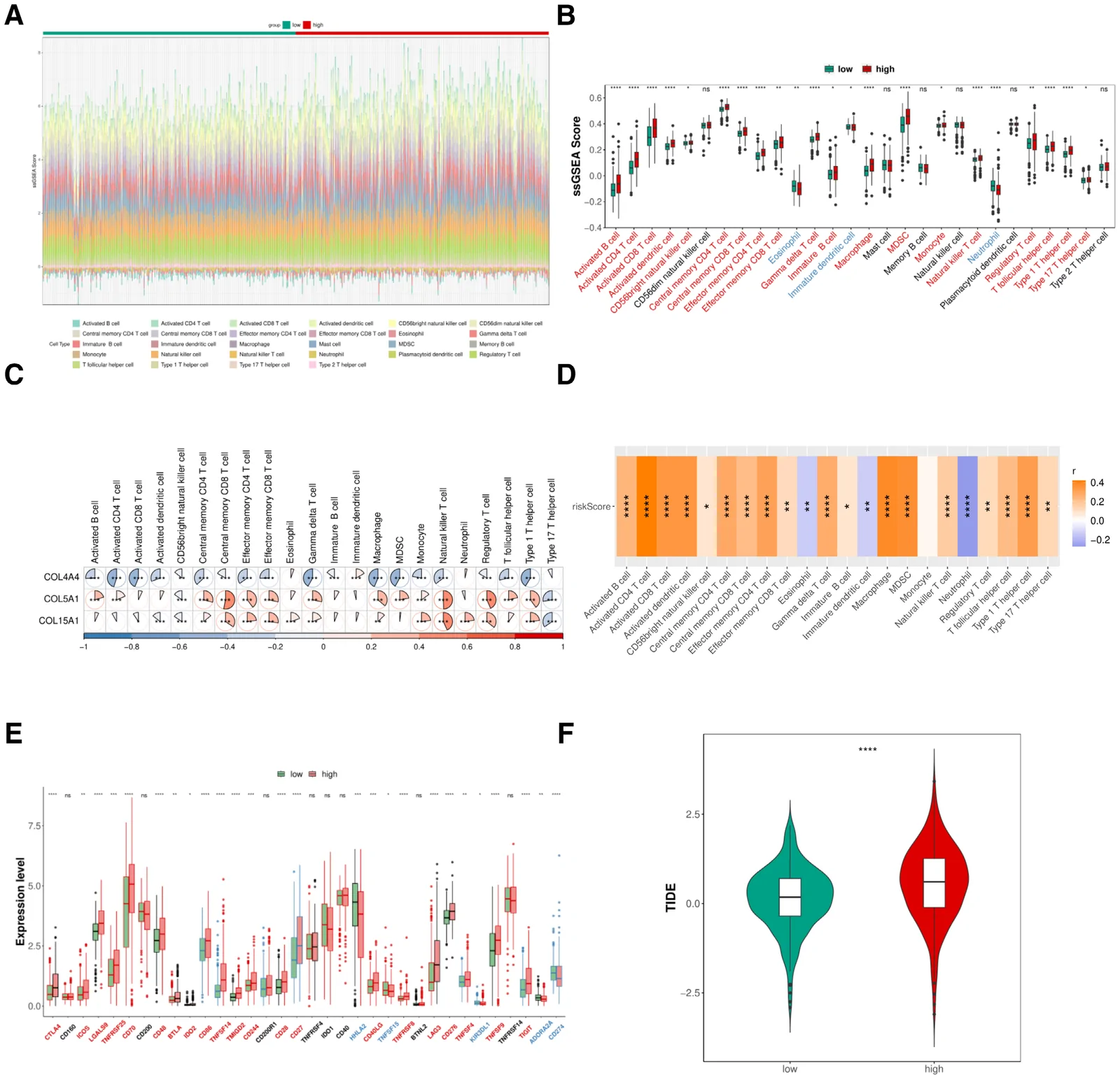
Immune infiltration in KIRC patient samples and its correlation with prognostic genes. Stacked plots (A) and box plots (B) illustrated variations in the infiltration patterns of 28 distinct immune cell types between the 2 risk groups. (C) Correlation between prognostic genes and immune cells. (D) Correlation between the risk score and immune cells. (E) Differences in immune checkpoints between the high and low-risk groups. (F) TIDE scores indicated a higher likelihood of immune evasion in the high-risk group.

Stacked plots (Fig. 7A) and box plots (Fig. 7B) demonstrated distinct immune cell infiltration patterns between the 2 risk groups, with significant differences observed in the immune infiltration percentages of 22 cell types. In the high-risk group, 19 immune cell types, including activated B cells, were significantly upregulated, while eosinophils, immature dendritic cells, and neutrophils showed significant downregulation. A heatmap of the correlation between 22 differentially infiltrated immune cells and the 3 prognostic genes (Fig. 7C) revealed strong associations: *COL5A1* was highly correlated with central memory CD8 T cells, *COL4A4* with CD4 T cells, and *COL15A1* with natural killer T cells (*P* < .001). The correlation analysis between these 22 differential immune cells and the risk score (Fig. 7D) showed inverse correlations for 3 immune cell types, while the remaining cells exhibited a direct correlation with the risk score. These findings highlight changes in the immune responses and microenvironment between the 2 risk groups, emphasizing the potential of modulating the immune microenvironment as a therapeutic strategy for influencing KIRC progression and offering new avenues for personalized treatments.

In the high-risk group, the expression of 21 common IC-related genes, such as *CTLA4*, *ICOS*, and *LGALS9*, was significantly upregulated, whereas 5 other checkpoint genes, including *HHLA2*, *TNFSF15*, and *KIR3DL1*, were downregulated (Fig. 7E). Additionally, TIDE scores showed significant differences between the groups (Fig. 7F), with the high-risk group demonstrating a greater likelihood of immune escape. These expression changes in IC-related genes likely influence KIRC’s immune surveillance and immune evasion mechanisms. Consequently, these differential IC-related genes may serve as critical therapeutic targets for KIRC treatment.

### 3.8. Prediction of therapeutic drugs

**Figure 8.**
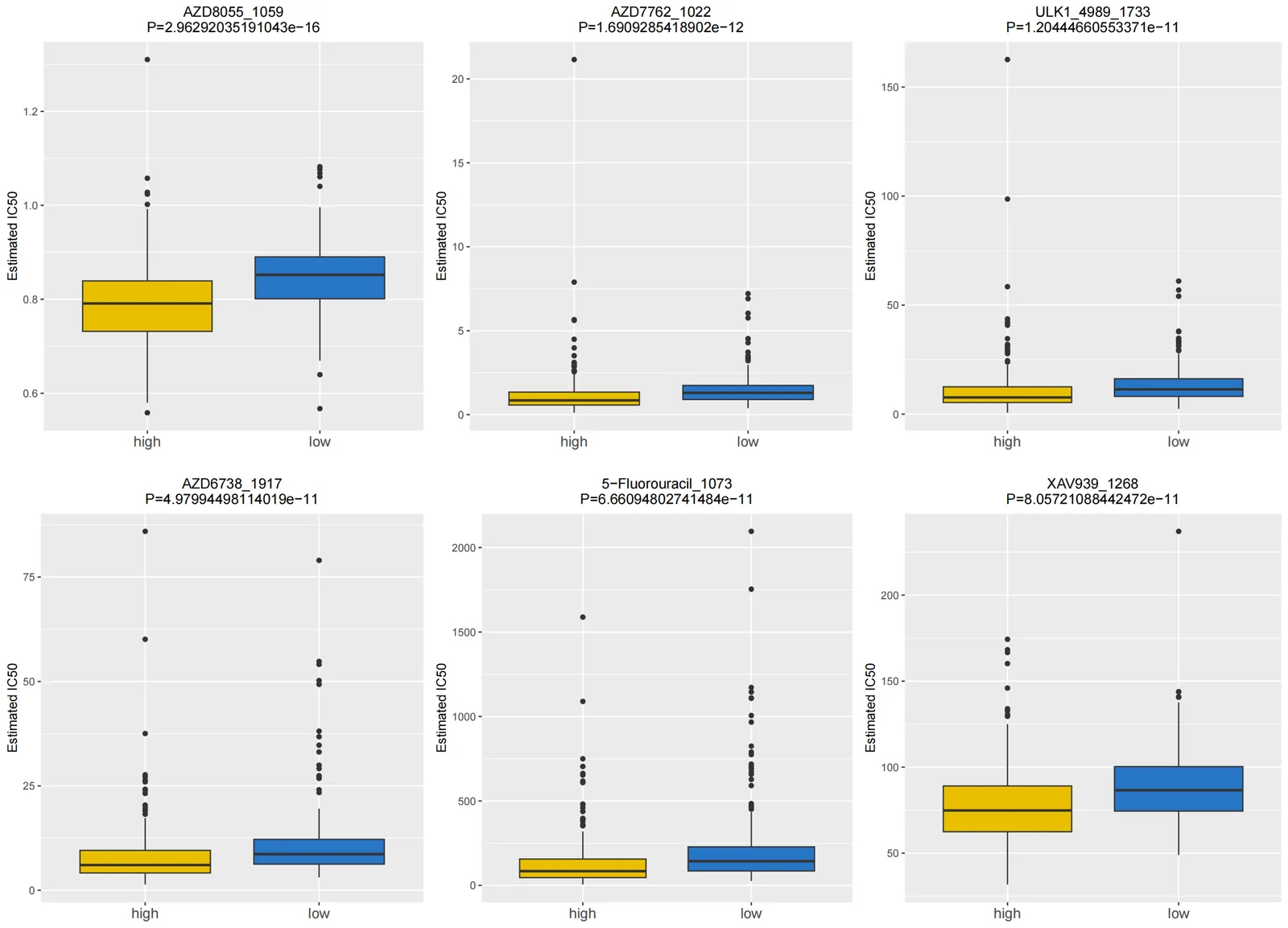
Significant differences in drug IC50 between high-risk and low-risk groups.

Among the 198 drugs analyzed, the IC_50_ values of 74 drugs, including AZD8055_1059, AZD7762_1022, ULK1_4989_1733, AZD6738_1917, 5-Fluorouracil_1073, and XAV939_1268, were significantly different between the 2 groups. The IC_50_ values in the high-risk group were higher, suggesting that drug efficacy may be influenced by the patient’s risk level (Fig. 8).

**Figure 9.**
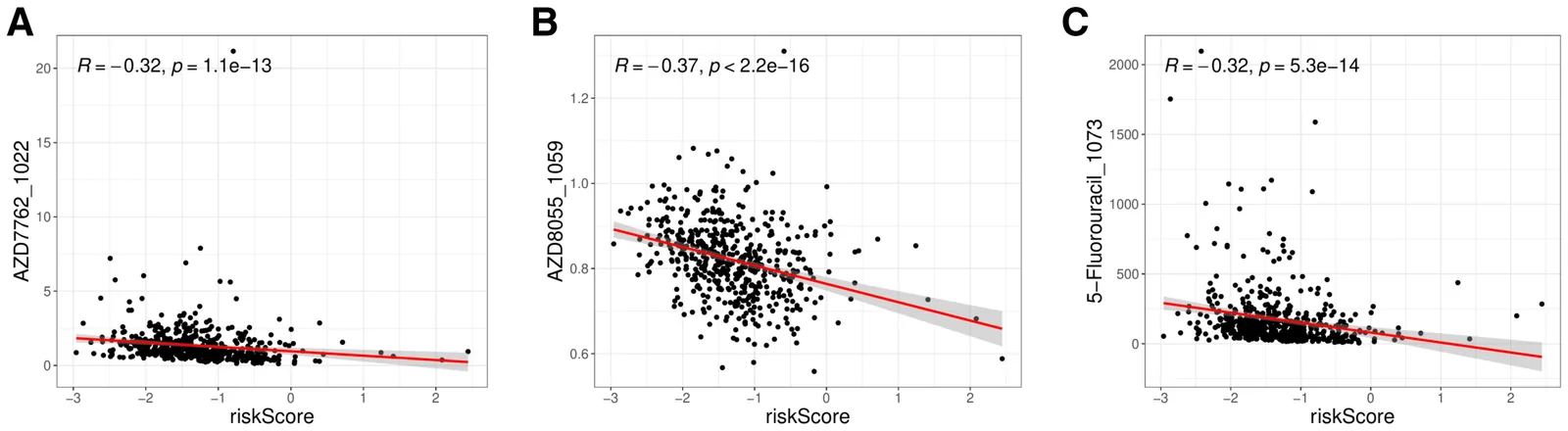
Drugs negatively correlated with risk scores. (A) AZD7762_1022. (B) AZD8055_1059. (C) 5-Fluorouracil_1073.

Furthermore, the IC_50_ values of 12 drugs were significantly correlated with the risk score. Notably, AZD7762_1022, AZD8055_1059, and 5-Fluorouracil_1073 exhibited negative correlations with the risk score (Figs. 9A-C). These results suggest that these drugs may be key in the treatment of KIRC, potentially offering therapeutic strategies tailored to patient risk profiles.

### 3.9. Regulatory network construction for prognostic genes

**Figure 10.**
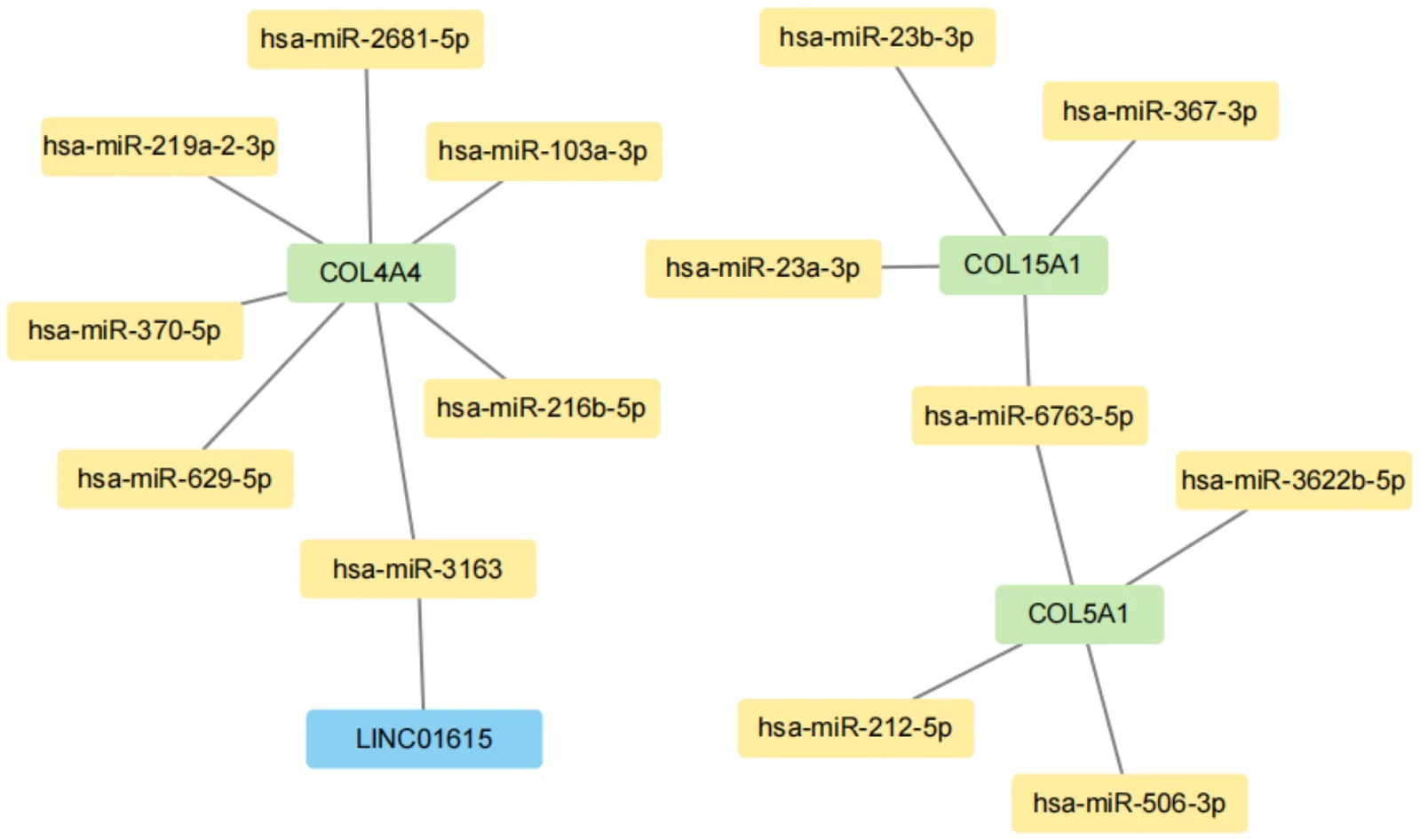
Regulatory network construction for prognostic genes.

Fourteen shared prognostic gene-associated miRNAs and 2163 shared miRNA-associated lncRNAs (including *LINC01615*) were predicted. The regulatory network involving lncRNA (*LINC01615*), miRNA, and mRNA (prognostic genes) is depicted in Figure 10, comprising 3 mRNAs (*COL4A4*, *COL5A1*, *COL15A1*), 14 miRNAs, and 1 lncRNA (*LINC01615*). In this network, the lncRNA regulates miRNAs, which in turn modulate mRNA expression. Specifically, *COL4A4* was associated with 7 miRNAs, while *COL5A1* and *COL15A1* were linked to 4 miRNAs each. These findings highlight a complex regulatory network involving lncRNAs, miRNAs, and prognostic genes. Notably, *LINC01615* regulates miRNAs such as hsa-miR-3163, which subsequently affects key prognostic genes like *COL4A4*. This highlights the critical role of *LINC01615* within the regulatory network and suggests its potential as a target for therapeutic strategies.

### 3.10. The expression of *COL5A1*, *COL15A1* and *COL4A4*

**Figure 11.**
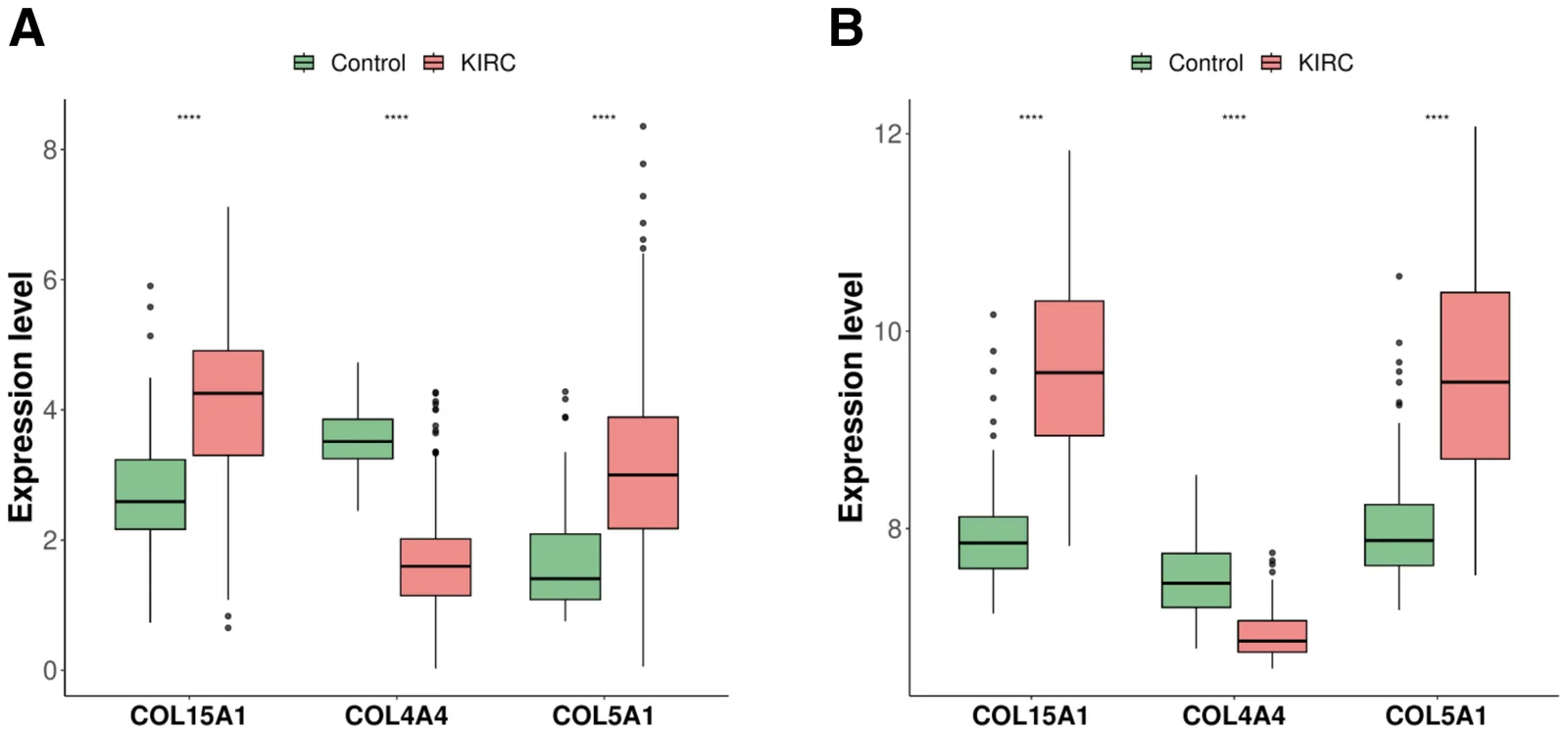
Expression of prognostic genes in the TCGA-KIRC and GSE40435 datasets. (A) TCGA-KIRC dataset. (B) GSE40435 dataset.

**Figure 12.**
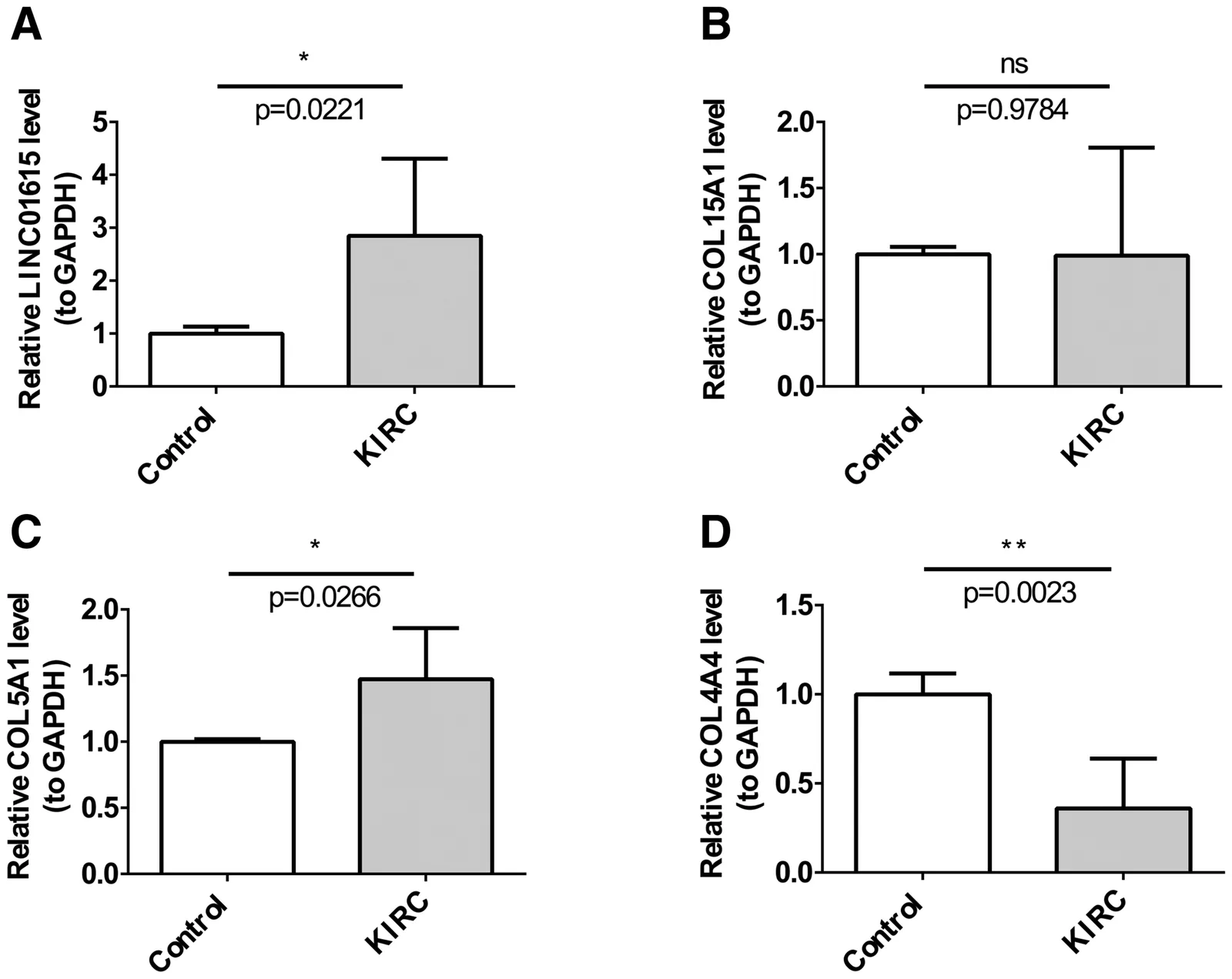
Expression of *LINC01615* and related genes in KIRC patients. (A) *LINC01615*. (B) *COL15A1*. (C) *COL5A1*. (D) *COL4A4*. *P < .05, **P < .01, ***P < .001, nsP > .05 KIRC = Kidney clear cell carcinoma.

In both the TCGA-KIRC and GSE40435 datasets, the expression trends of the prognostic genes were consistent and significantly different across the 2 datasets (Figs. 11A-B). *COL5A1* and *COL15A1* showed elevated expression in the KIRC group, while *COL4A4* was predominantly expressed in the control group. To assess the stability of these gene expressions in clinical samples, RT-qPCR was performed. Compared to the control group, the expression levels of *LINC01615* and *COL5A1* were significantly upregulated in the KIRC group, while *COL4A4* was notably downregulated (*P* < .05), consistent with the results observed in the datasets. However, no significant difference was observed in the expression of *COL15A1*, possibly due to the small sample size (Fig. 12). These differential expression patterns further reinforce the prognostic significance of these genes in KIRC.

## 4. Discussion

KIRC is the most common type of renal cancer in adults, with the majority of patients diagnosed at an advanced stage.^[39]^ Currently, there are no highly specific biomarkers for early diagnosis or treatment, contributing to a relatively poor prognosis. *LINC01615* has been identified as an oncogene in various cancers, influencing tumor cell behavior.^[40,41]^ However, its role in the development and progression of KIRC, as well as its prognostic significance, remains unclear. This study provides a comprehensive analysis of the value of *LINC01615* in KIRC, incorporating differential expression analysis, PPI network construction, and Cox regression analysis. Three prognostic genes related to *LINC01615*: *COL4A4*, *COL5A1*, and *COL15A1*: were identified, and a risk model was established. ROC analysis was performed to assess the accuracy of the risk model, confirming age and risk score as independent prognostic factors. Finally, bioinformatics approaches such as GSEA enrichment and immune-related analysis were used to explore the underlying mechanisms responsible for the prognostic differences between high- and low-risk groups, offering valuable insights for prognostic diagnosis and immunotherapy in KIRC.

*COL4A4*, a member of the type IV collagen family, plays a pivotal role in the structure and function of basement membranes, particularly in the kidney, where it is involved in the tubular basement membrane.^[42]^ Variations in the COL4A3/*COL4A4*/COL4A5 genes have been found in 31.1% of patients with IgAN-tGBM (sporadic IgA nephropathy [IgAN] with thinned glomerular basement membrane injury), suggesting that mutations in the COL4 genes contribute to the tGBM phenotype in sporadic IgAN.^[43]^ In KIRC, *COL4A4* expression is generally low, and this reduced expression correlates with lower clinical stage, pathological stage, and WHO grade.^[44]^ As a protective factor, *COL4A4* may serve as a potential therapeutic target for KIRC.^[45]^ However, the direct role of *COL4A4* in KIRC has not been thoroughly investigated, and further research is necessary to determine whether it influences tumor cell migration and metastasis by regulating the structural integrity of the tubular basement membrane.

The *COL5A1* gene encodes the α1 polypeptide chain of type V collagen,^[46]^ primarily located in the ECM, where it plays a critical role in tissue support and structural maintenance. *COL5A1* is overexpressed in various cancers and is associated with tumor progression and poor prognosis. For example, studies have shown that *COL5A1* promotes the development and progression of triple-negative breast cancer by facilitating crosstalk between tumor cells and macrophages.^[47]^ Silencing *COL5A1* expression using siRNA significantly suppresses tumor growth *in vivo*, suggesting its involvement in the malignant progression of glioma.^[48]^ Additionally, *COL5A1* has been identified as a co-expressed gene with LOX, playing a critical role in KIRC.^[49]^ Experimental evidence also demonstrates *COL5A1* overexpression in renal clear cell carcinoma, and its downregulation significantly inhibits renal cancer cell proliferation.^[50]^ In vitro experiments reveal that reduced *COL5A1* expression induces apoptosis and inhibits migration and invasion of renal cancer cells.^[51]^ These findings confirm that *COL5A1* is a risk factor for KIRC and is highly expressed in tumor samples, supporting its prognostic value in KIRC.

*COL15A1* encodes the α chain of type XV collagen, which is widely distributed in tissues, with the highest expression in the basement membrane zone. It plays a key role in anchoring the basement membrane to the underlying connective tissue matrix.^[52]^ *COL15A1* possesses anti-angiogenic and antitumor properties.^[53]^ Its interaction with P4HB regulates the growth and malignancy of HepG2.2.15 cells, suggesting a potential anticancer role in liver cancer.^[54]^ A pan-cancer study revealed elevated expression of *COL15A1* in KIRC, consistent with the findings of this study,^[55]^ although its precise function in KIRC still requires further investigation.

This study found that all the genes related to the prognosis of KIRC are collagen-related genes. Their abnormal expression may disrupt the integrity of the basement membrane, allowing tumor cells to break through the barrier and enhance their invasive and metastatic abilities, thereby promoting the progression of KIRC.^[56]^ Moreover, in lung cancer cells, overexpression of *LINC01615* promotes EMT, while knockdown inhibits it.^[13]^ Given that collagen is a key component of the ECM, it is speculated that *LINC01615* may regulate the composition and structure of the ECM by influencing the transcription of prognosis-related genes, thereby modulating the migration and invasion of tumor cells. Further research has revealed that *LINC01615* has a direct targeting relationship with miR-185-5p,^[57]^ miR-185-5p can regulate collagen metabolism; increased expression can induce collagen production and pro-fibrotic activation in cardiac fibroblasts, while inhibition weakens this effect.^[58]^ Its mimics can also increase the levels of α-smooth muscle actin, fibronectin, collagen I, and collagen III in HK-2 cells.^[59]^ We speculate that in the KIRC environment, *LINC01615* may indirectly affect the expression of prognostic genes (*COL4A4*, *COL5A1*, and *COL15A1*) through interaction with miR-185-5p, influencing collagen expression and ECM remodeling, and ultimately affecting the invasive and metastatic phenotype of KIRC. However, there is currently a lack of direct experimental evidence regarding how *LINC01615* specifically regulates these prognostic genes (such as *COL4A4*) in KIRC, as well as the mechanism by which it enhances the migratory ability of KIRC cells by disrupting the integrity of the basement membrane. Therefore, future research still needs to conduct experimental verification in KIRC models.

GSEA enrichment analysis in this study identified several key pathways potentially involved in KIRC development, including complement and coagulation cascades, cytokine-cytokine receptor interaction, fatty acid metabolism, ribosome function, and the degradation of valine, leucine, and isoleucine. Mounting evidence has confirmed that KIRC is essentially a metabolic disease driven by global energetic metabolic reprogramming.^[60–63]^ Glycolytic metabolic flux is drastically rearranged within KIRC cells,^[64–66]^ accompanied by impaired mitochondrial bioenergetics and oxidative phosphorylation (OxPhox), as well as widespread dysregulation of lipid metabolism.^[65,67]^ Metabolic reprogramming also serves as a core regulator of tumor cell drug sensitivity and chemoresistance,^[68,69]^ and controls multiple malignant biological properties of renal cancer stem cells.^[70,71]^ In addition, activated metabolic signaling cascades can modulate angiogenesis and inflammatory signatures within tumor lesions.^[72,73]^ Consistent with these published findings, our GSEA results exhibited prominent enrichment of fatty acid metabolism pathways, further validating the central role of lipid metabolic disorders in KIRC pathogenesis. The collagen-based risk signature constructed in this study may indirectly mirror metabolic heterogeneity among KIRC patients, which partially explains the distinct drug response differences between high-risk and low-risk subgroups observed in our drug sensitivity analysis.

Imbalance in the coagulation and anticoagulation systems in cancer individuals induces a hypercoagulable state, contributing to tumor progression.^[74]^ Furthermore, the fatty acid metabolism pathway-related gene ACADM has been recognized as a biomarker for KIRC, influencing tumor development by inhibiting fatty acid β-oxidation, leading to energy deficiency and abnormal lipid accumulation in KIRC cells.^[75]^ Abnormalities in the expression, mutation, and modification of ribosomal proteins contribute to tumor progression.^[76]^ For example, ribosomal protein S25 regulates the MDM2-p53 pathway, playing a critical role in cancer development and progression.^[77]^ These findings suggest that these pathways are pivotal in tumorigenesis, warranting further research to explore their specific roles in KIRC.

Immune infiltration analysis revealed a significant association between immune cells in KIRC, particularly activated CD4 T cells, central memory CD8 T cells, and natural killer T cells, and the prognostic genes *COL4A4*, *COL5A1*, and *COL15A1*. Xu et al demonstrated that in gastric adenocarcinoma, *COL4A4* significantly enhances the expression of activation markers on CD4 T cells,^[78]^ promoting their proliferation and differentiation. A pan-cancer analysis by Zhu et al found that tumors with high *COL5A1* expression exhibited decreased numbers and functionality of central memory CD8 T cells.^[79]^ Additionally, the absence of *COL15A1* may disrupt stromal integrity, influencing the infiltration and activation of NKT cells.^[53,54]^ These findings suggest that the identified prognostic genes may regulate the immune microenvironment by modulating the activity of immune cells, thus impacting tumor progression and treatment responses. Understanding these associations could contribute to the development of novel immunotherapeutic strategies to enhance KIRC treatment efficacy. This study offers valuable insights for the development of innovative immunotherapy approaches.

Notably, KIRC is recognized as one of the tumors with the most abundant immune cell infiltration.^[80,81]^ Comprehensive evidence indicates that overall tumor microenvironment characteristics dominate tumor biological behaviors and profoundly affect patients’ responses to systematic antitumor therapy.^[82–85]^ In the present study, we observed massive immune cell infiltration and elevated immune escape tendency in the high-risk cohort, which may be mediated by *LINC01615*. As a crucial lncRNA related to KIRC prognosis, *LINC01615* can modulate immune cell infiltration and shape tumor inflammatory and immune evasion phenotypes, which may partly interpret the differential IC expression and disparate immunotherapy efficacy between 2 risk groups. Combined with the above metabolic reprogramming features, the crosstalk between metabolic disorders and immune microenvironment remodeling driven by *LINC01615* may jointly accelerate KIRC progression and immune escape. Since this study only relies on bioinformatics mining without cellular or animal functional verification, the regulatory linkage between *LINC01615*, metabolic pathways and tumor immune microenvironment is merely a preliminary hypothesis and requires further experimental validation in follow-up studies.

This study found that AZD7762_1022, AZD8055_1059 and 5-FU_1073 were negatively correlated with the risk score, which implies that these drugs might be more effective in cancer patients with high-risk scores. AZD7762 is an effective CHK1/CHK2 inhibitor,^[86]^ which can reduce cisplatin-induced DNA damage by inhibiting the activation of the G2/M cell cycle checkpoint and regulating the Chk1-Cdc25C-Cdc2/Cyclin-B1 signaling pathway.^[87]^ Meanwhile, AZD7762 can also enhance the sensitivity of urothelial carcinoma cells to gemcitabine by inhibiting DNA repair mechanisms and interfering with cell cycle checkpoints.^[88]^ AZD8055 can induce G1 phase cell cycle arrest and apoptosis, and can also inhibit EMT and MMP9 expression, reducing the migration and invasion of bladder cancer cells, and to some extent, preventing the interaction between bladder cancer cells and macrophages.^[89]^ 5-Fluorouracil, as a major chemotherapy drug for colorectal cancer, has clinical efficacy that is often limited by drug resistance.^[90]^ The concurrent use of 5-Fluorouracil, which has been approved by the US Food and Drug Administration (FDA), and KIRC treatment drugs (such as sorafenib and everolimus) may help improve the therapeutic effect of down-regulating hsa-miR-200c-3p.^[91]^ Considering the cardiotoxicity caused by AZD7762,^[92]^ in actual clinical applications, the selection of appropriate drugs and treatment plans should be based on the specific conditions of patients. At the same time, combination therapy may be an effective way to improve therapeutic effects and overcome drug resistance. Future research should focus on exploring the synergistic mechanisms between different drugs, conducting more clinical trials to verify the efficacy and safety of combination therapy, and providing more effective and safer treatment options for KIRC patients with high-risk scores.

## 5. Conclusion

This study identified 3 prognostic genes (*COL4A4*, *COL5A1*, and *COL15A1*) associated with *LINC01615* in KIRC through bioinformatics analysis. Based on these genes, a risk model was constructed, which demonstrated good predictive performance. Combined with age, N stage and risk score, the nomogram provided a personalized prognostic assessment tool for clinical use. Additionally, high-risk patients showed an enhanced tendency for immune escape, and the risk score was negatively correlated with the sensitivity to specific drugs (such as 5-Fluorouracil_1073). These results provided important evidence for screening potential beneficiaries of immunotherapy and guiding individualized medication. However, only 5 clinical samples were used in the current RT-qPCR, with a small sample size, which might affect the statistical significance and generalizability of the results. Therefore, it is necessary to expand the sample size in future studies to confirm the validity and universality of these findings. Meanwhile, a series of functional experiments were conducted to explore the specific mechanism of action between *LINC01615* and the related prognostic genes, as well as their key roles in the pathogenesis of clear cell renal cell carcinoma (KIRC). Moreover, a large amount of evidence-based medical evidence is needed to verify the predictive ability of the prognostic risk model and the clinical application value of the discovered therapeutic drugs.

## Acknowledgments

We are grateful to all authors for their contributions to this study.

## Author contributions

**Conceptualization:** Shijie Guo, Yubin Wang.

**Software:** Shijie Guo, Shuangqin Xu.

**Visualization:** Shijie Guo, Shuangqin Xu.

**Data curation:** Shuangqin Xu.

**Formal analysis:** Peng Song, Jinshan Fu.

**Methodology:** Shengxing Wang, Wengui Xie.

**Funding acquisition:** Yubin Wang.

**Investigation:** Yubin Wang.

**Project administration:** Yubin Wang.

**Resources:** Yubin Wang.

**Supervision:** Yubin Wang.

**Validation:** Yubin Wang.

**Writing – original draft:** Shijie Guo, Shuangqin Xu.

**Writing – review & editing:** Shijie Guo, Shuangqin Xu, Yubin Wang.




