# Supplementary material for: Prognostic and immunological value of LINC01615-related genes in kidney clear cell carcinoma: Integrated bioinformatics analysis and expression validation
Source: Medicine (Baltimore). 2026 Aug 21;105(34):e50176. doi: 10.1097/MD.0000000000050176 (PMC13502803; doi:10.1097/MD.0000000000050176)
Supplement: Supplementary file 1 [file medi-105-e50176-s001.docx]

| **Primers** | **Sequences (5’ to 3’)** | |
| --- | --- | --- |
| LINC01615-F | CATCCAACAACAAGCCTGCG | |
| LINC01615-R | GCAAGGGGTTCTTCCAAGGT | |
| COL15A1-F | GGACTGTTGTCCACCTACCG |  |
| COL15A1-R | CTGGGGCCAAGAAGGATCTG |  |
| COL5A1-F | GACAAGAAGTCCGAAGGGGC |  |
| COL5A1-R | GGTAGGTGACGTTCTGGTGG |  |
| COL4A4-F | GCGCTCCCTAGGATGGGATT |  |
| COL4A4-R | GGCCCATCAGGACCTCTTTC |  |
| GAPDH-F | CGAAGGTGGAGTCAACGGATTT |  |
| GAPDH-R | ATGGGTGGAATCATATTGGAAC |  |

Supplementary Table 1 Primers of prognostic genes, LINC01615 and internal reference gene (GAPDH)
